# Mistrial or Misdiagnosis: The Importance of Autopsy and Histopathological Examination in Cases of Sudden Infant Bronchiolitis-Related Death

**DOI:** 10.3389/fped.2020.00229

**Published:** 2020-05-27

**Authors:** Giuseppe Bertozzi, Francesca Maglietta, Benedetta Baldari, Livia Besi, Alessandra Torsello, Cira Rosaria Tiziana Di Gioia, Francesco Sessa, Mariarosaria Aromatario, Luigi Cipolloni

**Affiliations:** ^1^Department of Clinical and Experimental Medicine, Section of Legal Medicine, University of Foggia, Foggia, Italy; ^2^Department of Anatomical, Histological, Forensic and Orthopedic Sciences, Sapienza University of Rome, Rome, Italy; ^3^Department of Radiological, Oncological and Pathological Sciences, Sapienza University of Rome, Rome, Italy

**Keywords:** sudden unexpected infant deaths (SUIDs), sudden infant death, autopsy, immunohistochemistry, acute viral bronchiolitis

## Abstract

Pediatrics, among all the branches of medicine, is a sector not particularly affected by a high number of claims. Nevertheless, the economic value of the compensation is significantly high, for example, in cases of children who suffered multiple disabilities following perinatal lesions with a long life expectancy. In Italy, most of the claims for compensation concern surgical pathologies and infections. Among these latter, the dominant role is taken by respiratory tract infections. In this context, the purpose of this manuscript is to present a case series of infant deaths in different emergency-related facilities (ambulances, emergency rooms) denounced by relatives. Following these complaints, the autopsy was performed, and subsequent histological examinations revealed the presence of typical and pathognomonic histological findings of acute viral bronchiolitis, whose morphological appearance is poorly reported in the literature. The analysis of these cases made it possible to highlight the following conclusions: the main problems in diagnosing sudden death causes, especially in childhood, are the rapidity of death and the scarce correlation between the preexistent diseases and of the cause of death itself. For all these reasons, the autopsy, either clinical or medicolegal, is mandatory in cases of sudden unexpected infant death to manage claim requests because only the histological examinations performed on samples collected during the autopsy can reveal the real cause of death.

## Introduction

Pediatrics, among the branches of medicine, is not particularly affected by a high number of claims. Nevertheless, the economic value of compensation is significantly high, for example, in cases of children who suffered multiple disabilities following perinatal lesions with a long life expectancy. According to Carroll and Buddenbaum (1) and Moriani et al. (2), examining the data collected

by an association of several American insurance companies (Physician Insurers Association of America), it was found that only 28% of the cases resulted in compensation; among these, in cases where no damages had been paid, the average cost per the only defense was $28,779, while it results in $67,502 for paid claims (3). The medical diagnoses, most commonly involved in civilian pediatric trials in the United States, were brain damage (average damages $440,379) and meningitis ($437,423). Respiratory problems in newborns account for $270,607. In Italy, data from the insurance company CARIGE Spa, in the period 2005–2012, excluding neonatology, highlighted how the main pathologies for which legal action was referred to surgery (gastrointestinal and testicular) and infections (more respiratory ones). Moreover, the data on litigation have also shown a different stratification of the number of requests for compensation, which were greater in the north and minors in central Italy, mostly involving the public health system (4). According to the Italian study performed during the 2005–2010 period, neonatology has also shown an overlapping geographical stratification, with the greatest interest for the public sector. The claims for damages following death, concerning the neonatal intensive care unit (NICU), mainly concern respiratory diseases (30.7% of cases) (5, 6). Both in the pediatric population in general, but especially in the neonatological one, the dominant role is taken by the respiratory tract infections.

In this context, the purpose of these case series is to demonstrate how the identification of the correct cause of death in the sudden unexpected infant deaths (SUIDs) allowed evaluating the absence of medical liability. Particularly, the definition of gold standard methods in similar cases could be considered very important to avoid the compensation in unjustified claim requests.

## Case Series

All procedures performed in the study were in accordance with the ethical standards of the institution and with the 1964 Helsinki Declaration and its later amendments or comparable ethical standards. Written informed consent was obtained from the first-degree relatives.

### Case 1

**Figure 1:**
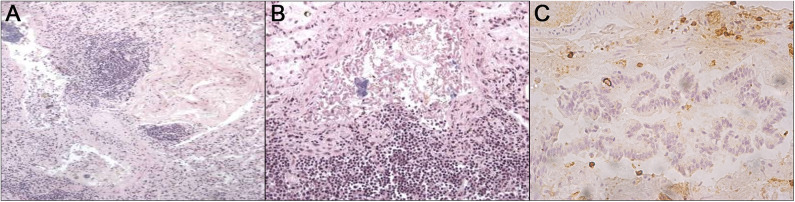
From left to right: **(A)** HandE staining let a leukocyte bronchial and peribronchial infiltrate be noticed; **(B)** HandE staining shows the bronchial walls with epithelial cells fallen into the lumen which appears filled by abundant amorphous eosinophilic material in which leukocytes can be detected; **(C)** immunohistochemical staining with anti-CD45 reveals peribronchial positivity.

A 10-month-old male infant died during Emergency Medical Services (EMS) transport to the hospital. When parents had been asked for any modification in their child habits, a mild “rhinitis” for a few days was told. For this reason, they went to their trusted pediatrician 2 days earlier, who suggested saline nasal rinses and a short turn check. The medical examiner documented no relevant external sign to explain death. Therefore, the parents sued the pediatrician for both penal and civil liability. During the forensic autopsy, the macroscopic examination was unremarkable except for mild edema affecting both lungs. On the contrary, histological examination showed in both lungs a diffuse transmural inflammation in the bronchiolar wall. Other tissue sections showed chronic inflammation, and bronchiolar wall fibrosis primarily restricted to bronchioles (Figure 1).

### Case 2

**Figure 2:**
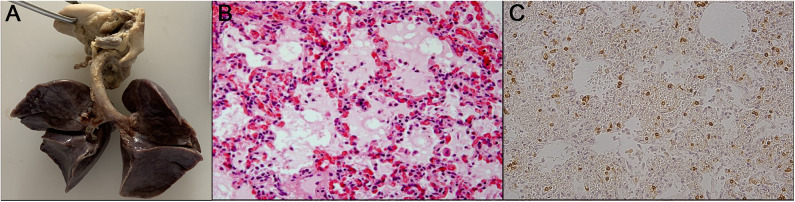
From left to right: **(A)** the not significant macroscopic study; **(B)** histological study; **(C)** immunohistochemistry shows the positivity to anti-CD45 staining. HandE staining shows considerable thickening of the septal structures due to the presence of abundant cellularity, mainly consisting of plasma cells and small lymphocytes. The alveolar spaces are atelectatic in some fields; in others, they show amorphous eosinophilic material inside. In many fields, bronchial and peribronchial infiltrates, consisting of lymphocytes and plasma cells, cover the bronchial structure itself.

An ambulance was called for a 9-month-old female infant, who lived in a nomad camp; her parents referred that suddenly she did not respond to external stimuli. Relatives did not refer to any symptoms neither clinical signs in the previous days. During the resuscitation maneuvers in place, the infant died. Thus, the prosecutor ordered the autopsy for alleged medical liability; her parents demanded the civil compensation to the local health insurance, thinking that during the ambulance transportation, there was medical responsibility. The macroscopic examination, both at the external corpse and internal organs, only showed severe pulmonary edema. The histology was characterized by lymphocytic infiltration of the bronchioles (Figure 2).

### Case 3

**Figure 3:**
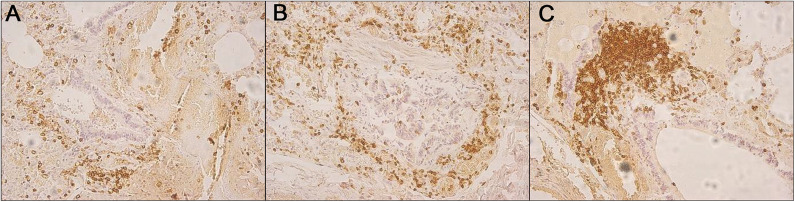
**(A–C)** Immunohistochemical staining CD45-positive showing peribronchial and intrabronchial wall leukocyte infiltrates.

An 18-month-old female infant was admitted to the emergency room of a pediatric hospital for severe cough and pharyngitis; she died after a few hours. Symptoms onset occurred the day before hospitalization. She was a preterm infant (29.3 weeks, birth weight 1,400 g) who suffered from severe respiratory distress at birth (Apgar score 1′ = 4) and needed a long period of hospitalization. After discharge, she showed neurodevelopmental impairment; moreover, a month before death, she suffered from many viral infective pathologies such as influenza and mononucleosis: all pathologies were successfully treated with standard pharmacological therapies. In this case, the judicial authority disposed of the forensic examination, suspecting medical liability to clarify penal and civil aspects: indeed, at the time of death, a claim for damages has been made to the hospital by the family of the patient. The autopsy showed congestion of tracheal and bronchial mucosa. At histological examination, focal edema and diffuse congestion of both lungs, acute emphysema, and peribronchial and intrabronchial wall leukocyte infiltrates were found; the same results involved nearby septal vessels (Figure 3).

## Materials and Methods

The method used in these cases, as in all cases of sudden death, consists of a rigorous and multidisciplinary methodological approach (7, 8):

- anamnestic collection and clinical findings: the clinical symptomatology presented by the subject before his death or in close chronological concurrence, clinical history of the case, and previous medical records;
- anatomical evidence: a macroscopic examination of all organs, their weight, consistency, and color at the autopsy, such as the appearance of fluids (blood, urine, vitreous humor).
- histomorphological examination H&E of the organ samples to study any alteration due to pathological condition;
- immunohistochemistry: to evaluate, in particular, the presence and the location of the main white blood cells *via* antibody anti-leukocyte common antigen (CD45).

The adherence to this diagnostic procedure not only allows you to check and evaluate a larger quantity of data but allows a complete evaluation on all fronts, from the study of which can confirm the suspicion and/or an unexpected result but crucial for investigations.

## Discussion

In the discussed cases, following both the autoptic and especially the microscopic examination, the cause of death was identified in all investigated cases: a rapidly progressive acute bronchiolitis was ascertained. These findings allowed exonerating doctors from any penal liability. The bronchiolitis was defined in 2006, from a collaboration between the American Academy of Pediatrics (AAP) and the European Respiratory Society (ERS), as “a constellation of clinical symptoms and signs including a viral upper respiratory prodrome followed by increased respiratory effort and wheezing in children <2 years of age” (9). Acute viral bronchiolitis (AVB) is a lower respiratory tract infective disorder, typically affecting infants <2 years old (90% of cases). Respiratory syncytial virus (RSV) is involved in up to 70% of cases, followed by rhinovirus (up to 25%); the remaining cases are related to coronavirus, adenovirus, influenza, and parainfluenza virus, and human metapneumovirus. Coinfections are common (10). However, the seasonality of bronchiolitis, generally more frequently found during the winter months, coincides with the seasonal pattern of RSV diffusion (11).

The infection starts in the upper respiratory tract, spreading to the lower airways in a few days. The bronchiolar damage is determined by the direct action of the virus on the epithelium of the same tract; alternatively, it was indirectly immune-mediated, and it was characterized by a peribronchial infiltration of white blood cell types, mainly mononuclear cells, with edema of the submucosa and adventitia (9). The pathophysiological continuation is caused by a mixture of edema, increased production of mucus, and progressive damage of the epithelium even to necrosis, which determines obstruction of the airflow, entrapment of distal air, atelectasis, and alteration of the ventilation/perfusion. The results are hypoxemia and increased respiratory work, which in turn worsens hypoxemia (9).

The most important extrapulmonary symptoms involve the brain (apnea, epileptic status) and heart (ventricular tachycardia, ventricular fibrillation, cardiogenic shock, complete heart block, and pericardial tamponade) and are common in children with severe infections (12).

The most dreadful complications of bronchiolitis are central apnea, a respiratory pause with bradycardia, cyanosis, pallor, and hypotonia that often requires hospitalization (13). Bronchiolitis represents a disease with high morbidity but low mortality. Death from respiratory failure in bronchiolitis is rare and varies from deaths from 2.9:100,000 in the UK to 5.3:100,000 in the US, for children under 12 months, with a relationship that goes hand in hand, reducing itself to the improvement of good intensive practices (9, 14–16).

In all these cases, in the absence of the clinical-anamnestic data that can guide the clinical diagnosis, the external examination data and the autopsy macroscopic data could point toward a diagnosis of SUID. In the case of Özdemir et al. (17), in fact, on the totality of the cases of malpractice claims, 57.5% of the children had died and 59.3% were subjected to autopsy. In these cases, the causes of death reported before and after the autopsies were different in 68%, and the medical staff was found to be responsible for 46.1% of the claims.

Therefore, the determination of the exact cause of death assumes fundamental importance to ascertain the causal link of any conduct of both health facilities and individual professionals in determining death (18–20). This allows not only a measurement of the quality of care provided by promoting public trust for the health system but also as a measure of clinical governance; moreover, it is possible to better manage the medicolegal disputes as a guarantee of ascertaining the truth. Indeed, in Italy, the data on the frequency of adverse events (AEs), preventable adverse events (PAEs), and negligent adverse events (NAEs) are available; nevertheless, data about malpractice claims are not available both under the penal and civil points of view. Furthermore, the epidemiological purposes cannot be forgotten, considering that it is the only reliable method of data collection. Indeed, a complete methodological approach, integrating clinical data, autopsy, and histological findings could be considered the best way to solve similar cases. In fact, in the reported case studies, histopathologic diagnostics identified pathognomonic signs of acute bronchiolitis characterized by edema, congestion, leukocytic infiltration in the bronchiolar wall, leukocytes in the peribronchial interstitial pulmonary space, allowing the identification of the exact cause of death. Therefore, these pieces of evidence have allowed excluding the medical responsibility in the reported cases, demonstrating that there are events not related to the supplied health care.

## Conclusion

The analysis of the presented cases shows that the autopsy is mandatory in SUID occurrence, in which the absence of anamnestic data and/or acute clinical signs does not allow to identify the cause of death. Hypothesizing medical negligence in each case, the autopsy was performed following the judicial appointment after the relative's complaint.

The subsequent histological examinations revealed the presence of typical and pathognomonic histological findings of AVB, whose morphological appearance is poorly described in the literature. Only the postmortem examinations have allowed excluding medical liability and therefore the compensation for damage.

In light of these findings, it could be considered essential an accurate evaluation of similar cases, collecting all data to avoid compensation in unjustified claims made against the hospital. In this way, it is possible to contain the hospital costs related to this kind of accident. For all these reasons, the autopsy combined with the subsequent examination represents a gold standard method to identify the absence of the hospital's responsibility in SUID cases.

## Data Availability Statement

All datasets generated for this study are included in the article/supplementary material.

## Ethics Statement

Written informed consent was obtained from the first-degree relatives for the publication of this case report.

## Author Contributions

GB, FM, MA, and LC contributed to the conception of the study and wrote the manuscript. FS, BB, LB, AT, and CD contributed significantly to literature review and manuscript preparation. GB, FM, FS, BB, LB, AT, CD, MA, and LC helped perform the analysis with constructive discussions and approved the final version.

## Conflict of Interest

The authors declare that the research was conducted in the absence of any commercial or financial relationships that could be construed as a potential conflict of interest. The handling editor declared a shared affiliation, though no other collaboration, with the authors GB, FM, BB, LB, AT, CD, FS, MA, and LC.
